# Parasitic infections in the organic beef cattle herds of southern Poland during the grazing season, with the first record of *Calicophoron daubneyi* (Dinnik, 1962) in the country

**DOI:** 10.2478/jvetres-2025-0012

**Published:** 2025-03-11

**Authors:** Paweł Nosal, Jerzy Kowal, Marta Basiaga, Andrzej Węglarz

**Affiliations:** Department of Zoology and Animal Welfare, University of Agriculture in Kraków, 30-059 Kraków, Poland; Department of Genetics, Animal Breeding and Ethology, University of Agriculture in Kraków, 30-059 Kraków, Poland

**Keywords:** helminths, paramphistomes, Strongylida, coccidia, beef cattle

## Abstract

**Introduction:**

Grazing cattle are vulnerable to the harmful effects of gastrointestinal parasites. Organically farmed cattle are even more so because conventional antiparasitic treatments are restricted, yet parasite infection patterns in Polish organic herds remain poorly documented.

**Material and Methods:**

Imported beef cattle were studied during the pasture season in four organic herds in southern Poland. The McMaster quantitative flotation method was used to estimate infection prevalence (P, %) coproscopically and to quantify intensities of coccidia oocyst output (Ic, OPG) and nematode egg output (In, EPG) per gram of faeces. The qualitative sedimentation method was applied to assess the presence of digenean eggs. Coccidial species of the *Eimeria* genus were identified by sporulation, and nematodes of the Strongylida order by larvoscopy. Digenean Paramphistomatidae were identified by morphological examination of adult fluke specimens obtained at slaughter from a sick heifer in one of the studied herds and by molecular analysis of the flukes’ internal transcribed spacer 2 ribosomal DNA.

**Results:**

The prevalence of *Eimeria* infection was P = 28.9 (23.8–34.5)%, and the mean Ic was 287 (113–793) OPG. Calves were most heavily infected, mainly with *E. bovis* and *E. zuernii*. The prevalence of nematode infections reached P = 46.0 (40.2–51.5)%, and the mean In was 113 (88–147) EPG. *Haemonchus placei* dominated over *Ostertagia* sp. and *Trichostrongylus axei*, and the most infected were first-time grazing yearlings. Paramphistome eggs were confirmed in only one herd. Morphological and PCR analysis of the adult rumen flukes revealed the presence of *Calicophoron daubneyi* (Dinnik, 1962) in this herd.

**Conclusion:**

This is the first Polish evidence of *C. daubneyi*, and it heralds an enhanced surveillance need regarding this highly pathogenic digenean.

## Introduction

Breeding beef cattle can be a good choice for grassland management in mountainous and foothill areas, where the basis of nutrition is pasture. When animal husbandry adheres to agroecological principles, it improves animal welfare, ensures high beef quality (the meat being referred to as “grass-fed beef”) and boosts production profitability. This mode of farming additionally contributes to improving the stability of agroecosystems, increasing biodiversity, improving soil fertility and protecting it from erosion, and increasing the attractiveness of rural areas to tourists. No beef cattle were bred in Poland until the 1990s, when large imports of meat cattle breeds from western European countries, the USA and Canada were initiated after the introduction of a programme for the development of beef cattle breeding. According to Statistics Poland, as of December 31, 2022 (https://stat.gov.pl), there were approximately 784,000 of these animals in the country, including about 135,000 suckler cows.

Beef cattle raised organically are particularly exposed to parasites, especially because no routine chemoprophylaxis is given to them while they graze. Infection with gastrointestinal parasites can lead to a condition known as parasitic gastroenteritis (PGE) in cattle of all ages, notably threatening first-season grazing calves. Although in most cases it occurs as a subclinical infection, the main impact of which is suboptimal production as a consequence of reduced feed intake and nutrient absorption, severe PGE can lead to weight loss and diarrhoea, and in some circumstances even to death. Bovine PGE is often coccidiosis, but mainly gastrointestinal nematode (GIN) species from the order Strongylida, such as *Ostertagia ostertagi* causing ostertagiosis, *Cooperia* spp., *Haemonchus placei, Trichostrongylus axei, Oesophagostomum radiatum*, and to a lesser extent *Bunostomum phlebotomum* and *Nematodirus* spp. constitute the principal causes of PGE (19, 28, 34).

The economic consequence of subclinical infections of coccidiosis is mainly poorer beef calf production (5, 29). According to Daugschies and Najdrowski (5), *Eimeria bovis, E. zuernii, E. alabamensis, E. auburnensis* and *E. ellipsoidalis* are thought to be pathogenic species in cattle because they cause the clinical signs of coccidiosis – typically watery or bloody diarrhoea followed by loss of appetite, depression, dehydration and weight loss, leading to retarded growth. Of these, particularly *E. bovis* and *E. zuernii* have a major impact worldwide because of their high virulence, especially in terms of the frequent mortality of calves under one year of age which they cause (42).

The predominance of coccidia and nematode infection on cattle farms is mainly related to their direct life cycle. However, some parasites with indirect life cycles, such as cestodes and trematodes, are also commonly found in grazing cattle around the world. The anoplocephalid tapeworms *Moniezia* spp. (in cattle mainly *M. benedeni*) use oribatid mites (Acari: Oribatida) as intermediate hosts and these cestodes are generally considered non-pathogenic unless present in very large numbers, especially in calves, where they can cause loss of growth efficacy (16). As for digeneans, recent studies confirm the greater prevalence of rumen paramphistomes over *Fasciola hepatica* (a liver fluke) in Europe (15). The intermediate hosts for *Paramphistomum* spp. are typically planorbid aquatic snails, whereas *F. hepatica* and the most frequently recorded rumen fluke in northern, southern and western Europe today – *Calicophoron daubneyi* (Dinnik, 1962), syn. *Paramphistomum daubneyi* – share the same key host, the mud snail *Galba truncatula*. The clinical symptoms of paramphistomiasis include lethargy, dehydration, severe scour and submandibular oedema, which occur as immature parasites excyst and penetrate the duodenal mucosa while migrating towards the rumen, causing significant damage to tissues that may be mortal (20). Mature paramphistome infections have been associated with ruminal papillae atrophy and inflammation and ulceration at the point of fluke attachment (11), as well as negative impacts on production indices, such as milk yields, growth rates, cold carcass weights and fat coverage at slaughter (2).

Although *Calicophoron daubneyi* is now the dominant paramphistome in Europe and commonly found infecting cattle, sheep and goats in France, Spain, Portugal, Italy, the United Kingdom, the Republic of Ireland, Belgium, Netherlands, Germany and lately the Czech Republic (22), its presence in Poland had not yet been confirmed prior to this research. Instead, occurrences of *Paramphistomum cervi* and *Paramphistomum ichikawai* in cattle have been documented, *P. cervi* being the dominant and more frequently identified species (13, 28, 30). The same fluke species are also found in sheep and free-living ruminants (6, 45). However, Gundłach *et al*. (13) have already stated that it may be possible for other paramphistomes not yet detected in Poland to be brought into the country together with imported ruminants. Similarly, Pilarczyk *et al*. (30) pointed out that importing cattle without the obligatory parasitological examination may risk establishing new parasite species in the country.

Since imported breeds of beef cattle have been only rarely monitored parasitologically in Poland over the last few decades, the aim of this study was to identify the parasitic fauna of the cattle and to assess the level of infection in selected organic herds in order to obtain data enabling appropriate herd management.

## Material and Methods

The study was conducted in the grazing season of 2022 on four farms (A–D) rearing organic beef cattle located in the mountainous and foothill areas of the Małopolska voivodeship of southern Poland (with centre points of 49°33′ N 21°13′ E and 49°35′ N 19°52′ E). The pastures were situated 550–600 m above sea level in the area of the western and central Beskids that form the mountain ranges within the outer western Carpathians.

The animals on all the examined farms were kept in deep-litter cowsheds, in a free-range system with unrestricted access to runs and pastures in the grazing season. The pasture quarters were partially enclosed with an electric fence and had numerous trees growing in them, providing protection against unfavourable weather conditions during grazing (*i.e*., strong sunshine, rain and wind) and enriching the forage base. After the animals finished grazing on a given plot, the undercrops were mown. The basis of the animals’ diet from May to October was pasture, but additionally, the animals had unlimited access to hay and mineral licks located in the barns (farms A–C) or runs (farm D). Running water was available *ad libitum* from troughs (or natural streams on farms A and C) in the pastures and from drinkers by the livestock buildings.

Farm A, with an area of 60 ha of grassland comprising meadows and alternate pastures, maintained a Limousine cattle herd of 75 animals, including 30 suckler cows and 2 breeding bulls. The stocking rate on this farm was 0.8 livestock unit (LU) per hectare. During the grazing season, the cattle were rotated through grazing quarters every three days, occupying an area of approximately 5 ha at a time.

Farm B herd consisted of 28 Limousine cattle, including 10 suckler cows and 1 breeding bull. During the year, 20 ha of grassland was used alternately as hay meadows or pasture. The quarters of approximately 1–2 ha were changed every 6–9 days at the beginning of the growing season, and from August, the herd had access to the entire pasture area. The stocking density was about 1.0 LU/ha.

Farm C kept Galloway cattle numbering 31 animals, with 12 suckler cows and 1 breeding bull. Again, grassland of 20 ha was used alternately, and the stocking rate was 0.9 LU/ha. Until August, the animals grazed for 7–10 days on a given quarter of approximately 1–2 ha, and thereafter they had access to the entire pasture.

Farm D maintained a herd of 50 Limousine cattle, there being 20 suckler cows and 1 breeding bull among them. The pasture covered an area of 40 ha, and the stocking rate was 0.9 LU/ha. Separate quarters of 3–4 ha each were grazed only for the first two months of the grazing season. Thereafter, the animals used the entire pasture area until the end of the growing season.

### Coproscopic examinations

#### Collection of faeces

No antiparasitic agents had been used in the herds for more than one year before the start of the study, and no animals showed clinical symptoms of any parasitic disease while sampling was taking place. Since rectal sampling was not possible, about 10 g of faeces was collected immediately after the defecation of any grazing animal was observed. To prevent sample contamination, only the top layer of freshly deposited excrement was collected using plastic gloves. Samples were stored at 4°C until laboratory examination could be carried out, which was within 24 h. A total of 287 samples were taken from May to October at monthly intervals.

#### McMaster quantitative flotation method and statistical analyses of parasitic infection

For the coproscopy, we applied the quantitative concentration McMaster technique in Roepstorff and Nansen’s modification (40), using a sucrose-saline saturated flotation solution with a specific gravity of 1.28. Briefly, 4 g of faeces were mixed with 56 mL of tap water, and subsequently 10 mL of the obtained uniform suspension was transferred into a test tube to be centrifuged for 5–7 min at 1,200 rpm. Afterwards, the supernatant was removed and faecal sediment resuspended in flotation fluid to a total volume of 4 mL, as a result of which the faecal suspension volume of 0.3 mL on a McMaster slide represented 1/20 g faeces, giving the method sensitivity of 20 eggs/oocyst per gram (EPG/OPG) of faeces. The presence of coccidial oocysts and helminth eggs was investigated under a Motic light microscope (Hong Kong, China) at 100× and 400× magnification using the keys of Taylor *et al*. (38) and Zajac and Conboy (44). Indistinguishable eggs of nematodes belonging to the Trichostrongylidae family (of the *Trichostrongylus, Ostertagia, Cooperia* and *Haemonchus* genera), the Chabertiidae family (*Chabertia ovina* and species of the *Oesophagostomum* genus) and the Ancylostomatidae family (*Bunostomum* sp.) were treated as eggs of the Strongylida order, whereas eggs of the *Nematodirus, Aonchotheca, Trichuris* and *Strongyloides* genera were morphologically identified, and the eggs of the *Moniezia* genus were recognised as being of the *M. benedeni* cestode species.

The prevalence of infection (P, % of animals infected) and the intensity of coccidial oocyst (Ic) or nematode egg (In) output as mean number per g of faeces of an infected host (EPG/OPG) were assessed and subjected to statistical comparisons. Statistical analyses were conducted using Quantitative Parasitology 3.0 software (35) designed to analyse the negative binomial distributions exhibited by the parasites. Fisher’s exact test was used to compare the prevalences of infection (P, %), and mean EPG/OPG values (In EPG/Ic OPG) were compared using the bootstrap one-way analysis of variance and the bootstrap *t*-test. The P and In/Ic measures of infection were given together with their 95% confidence intervals (CI), calculated according to Sterne’s method (Sterne and Blaker’s exact CI^P^) for prevalence, or by the bootstrap BCa method for population mean EPG/OPG values (CI^I^). Differences were considered significant when the P-value was less than 0.05, and highly significant when it was less than 0.001.

#### Sporulation of coccidial oocysts for identification of *Eimeria* species

In order to identify the coccidial oocysts of the *Eimeria* genus to species level with the use of the keys of Eckert *et al*. (7), pooled faecal samples from each herd and month were sporulated in 2% aqueous (w/v) potassium dichromate at room temperature for 10 d.

#### Larval culture for identification of Strongylida nematodes

Similarly, a coproculture was established according to Henriksen and Korsholm (14) from the bulk faecal samples of each herd and each month, and was incubated at room temperature for 10 d to obtain the infective third stage nematode larvae (L_3_). These were identified following isolation by the Baermann larvoscopic method on the basis of their morphometrical features according to van Wyk and Mayhew’s keys (41).

#### Qualitative sedimentation method

The qualitative sedimentation method according to Żarnowski and Josztowa (46) was used to confirm fluke infection in cattle. The remaining sediment with a small amount of water was stained with a 1% solution of malachite green on a watch glass and examined under a stereomicroscope (PZO, Warsaw, Poland) at 40× magnification for the presence of the heavy eggs of digenetic trematodes.

### Morphological examination of adult Paramphistomidae specimens

#### Origin of the host animal and material collection

A 1.5 year-old heifer in the herd on farm A in poor condition was selected for slaughter in September 2022, and additionally was necropsied to examine it for the presence of paramphistomes as a consequence of a rumen fluke infection revealed by the sedimentation method. From several thousand adult trematode specimens collected, rinsed and kept in 0.9% isotonic saline solution until being fixed in 70% ethanol, 15 were used for morphological examination and molecular analysis.

#### Morphological identification

Ten adult flukes were remowed from the ethanol, processed, stained and sectioned by hand for identification according to the technique described by Jones (17). The morphological features that differentiate the species (8, 9, 10) were analysed. All the adults included in the molecular study were examined morphologically and confirmed as paramphistomes.

### Molecular analysis of adult Paramphistomidae specimens

#### Genomic DNA isolation

Extraction of DNA from five flukes obtained from the slaughtered heifer was performed using the Genomic Mini AX Bacteria+ kit (A&A Biotechnology, Gdańsk, Poland) as specified by the manufacturer, with additional mechanical lysis of the sample in a FastPrep-24 homogeniser (MP Biomedicals, Irvine, CA, USA) using zirconium beads.

#### PCR amplification

The internal transcribed spacer 2 (ITS-2) ribosomal DNA (rDNA) and partial flanking 5.8 Svedberg unit (S) and 28S regions were amplified using the generic forward ITS-2Trem For (TGTGTCGATGAAGAGCGCAG) and reverse ITS-2Trem Rev (TGGTTAGTTTCTTTTCCTCCGC) trematode primers (20). A PCR was conducted using a 50 μL reaction volume containing 25 μL PCR Mix Plus HGC (high guanine-cytosine content) (A&A Biotechnology; composed of 0.1 U/μL Taq DNA polymerase, 4 mM MgCl_2_ and 0.5 mM deoxynucleotide triphosphates), 100 μM of each primer and 0.2 μL of genomic DNA. The PCR was run under the following conditions: 94°C for 2 min as the pre-denaturation step; 30 cycles of 94°C for 0.5 min denaturation, 58°C for 0.5 min annealing and 72°C for 1 min extension; and 72°C for 5 min as the final extension.

#### Sequence analysis

DNA fragments obtained from the amplification reaction were purified using the Clean-Up AX kit (A&A Biotechnology). The PCR products were suspended in 10 mM tris-HCl pH 8.0 buffer, diluted to a concentration of 50 ng/μL and sent for sequencing to Macrogen Europe (Amsterdam, the Netherlands). The final sequences were analysed with the CLC Main Workbench 22 package (Qiagen, Hilden, Germany), and compared with reference sequences in GenBank at the European Bioinformatics Institute website (http://www.ebi.ac.uk/), using the BLAST program and rRNA/ITS databases (http://blast.ncbi.nlm.nih.gov). The sequences generated in the study were submitted to GenBank.

## Results

### Coproscopic examinations

A 28.9% (23.8–34.5) proportion of the examined animals were infected with *Eimeria* spp., and the mean Ic was 287 (113–793) OPG (Table 1). Adult cows were statistically significantly (P-value < 0.001) less frequently infected than the younger age groups, and the highest level of infection (P = 83.3 (41.1–99.2)% and Ic = 1,268 (28–3,700) OPG) was observed in calves (Table 1). The coccidia infection prevalence among the cattle also differed statistically significantly between herds (P-value < 0.05), from 16.9 (9.7–27.4)% in the case of herd D to 39.0 (29.9–49.0)% in herd A, with the mean Ic ranging from 42 (29–53) OPG in herd B to 524 (59–2,790) OPG in herd C. The highest level of infection (P = 31.5 (20.2–45.3)% and Ic = 648 (74–3,480) OPG) was observed in the autumn month of October, the last month of grazing. Of the twelve species of *Eimeria* genus identified (Table 2), *E. bovis* or *E. zuernii* occurred most frequently, depending on the herd, both usually being detected more than *E. ellipsoidalis*. Other species had a share of from 0.2 (*E. brasiliensis*) to up to several percent in the coccidial species’ composition.

**Table 1.** Parasitic infection of 287 organic beef cattle examined copro-microscopically using the McMaster flotation method: levels by herd, animal age and month of the grazing season

		n	Index	*Eimeria* sp.	Strongylida	*Nematodirus* sp.	*Strongyloides* sp.	*Trichuris* sp.	*Aonchotheca* sp.	*Moniezia benedeni*
In total		287	P	28.9	46.0	5.2	2.1	1.4	1.0	4.2
			CI^P^	23.8–34.5	40.2–51.5	3.1–8.5	0.9–4.5	0.5–3.6	0.3–3.1	2.4–7.3
			Ic/In	287	113	177	53	35	20	++
			CI^I^	113–793	88–147	62–324	23–93	20–50	NC	(+)–(+++)
Herd	A	100	P	39.0^a^	44.0	6.0^ab^	3.0	1.0	0.0	5.0
			CI^P^	29.9–49.0	34.4–54.0	2.6–12.4	0.8–8.4	0.1–5.3	0.0–3.8	2.0–11.3
			Ic/In	297	56^b^	23^b^	60	80	NE	+++
			CI^I^	104–974	42–76	20–27	20–100	NC	NC	(+)–(+++)
	B	47	P	23.4^ab^	51.1	0.0^b^	0.0	2.1	2.1	2.1
			CI^P^	13.2–37.5	37.0–65.0	0.0–8.0	0.0–8.0	0.1–11.3	0.1–11.3	0.1–11.3
			Ic/In	42	48^b^	NE	NE	20	20	+
			CI^I^	29–53	33–91	NC	NC	NC	NC	NC
	C	69	P	30.4^ab^	44.9	11.6^a^	4.3	1.4	1.4	5.8
			CI^P^	20.3–42.7	33.2–57.3	5.4–21.6	1.2–12.1	0.1–7.7	0.1–7.7	2.0–14.3
			Ic/In	524	192^a^	313^a^	47	20	20	+
			CI^I^	59–2,790	125–306	118–498	20–67	NC	NC	(+)–(++)
	D	71	P	16.9^b^	46.5	1.4^b^	0.0	1.4	1.4	2.8
			CI^P^	9.7–27.4	35.1–58.5	0.1–7.5	0.0–5.3	0.1–7.5	0.1–7.5	0.5–9.7
			Ic/In	60	162^a^	20	NE	20	20	+
			CI^I^	25–118	106–241	NC	NC	NC	NC	NC
Age	Calves (<0.5 year)	6	P	83.3^e^	33.3	0.0	33.3^a^	0.0	0.0	0.0^ab^
			CI^P^	41.1–99.2	6.3–72.9	0.0–41.1	6.3–72.9	0.0–41.1	0.0–41.1	0.0–41.1
			Ic/In	1268	320	NE	110	NE	NE	NE
			CI^I^	28–3,700	40–320	NC	80–110	NC	NC	NC
	Yearlings (0.5–1 year)	13	P	69.2^e^	69.2	7.7	15.4^a^	0.0	7.7	23.1^a^
			CI^P^	41.3–88.7	41.3–88.7	0.4–34.2	2.8–43.4	0.0–22.5	0.4–34.2	6.6–52.0
			Ic/In	71	113	20	20	NE	20	+
			CI^I^	36–164	56–184	NC	NC	NC	NC	+
	Heifers (1–2 years)	40	P	52.5^e^	35.0	2.5	0.0^b^	0.0	0.0	0.0^b^
			CI^P^	37.2–67.7	21.2–51.3	0.1–13.3	0.0–8.4	0.0–8.4	0.0–8.4	0.0–8.4
			Ic/In	182	53	20	NE	NE	NE	NE
			CI^I^	32–486	33–100	NC	NC	NC	NC	NC
	Cows (>2 years)	228	P	21.1^f^	46.9	5.7	0.9^b^	1.8	0.9	3.9^b^
			CI^P^	16.2–26.9	40.3–53.5	3.2–9.6	0.2–3.2	0.6–4.5	0.2–3.2	2.0–7.4
			Ic/In	270	117	202	30	35	20	++
			CI^I^	63–1,270	88–159	77–368	20–30	20–50	NC	(+)–(+++)
Month	May	51	P	27.5	70.6^a^	0.0	3.9	0.0	3.9	0.0
			CI^P^	16.4–41.1	56.9–81.6	0.0–7.3	0.7–13.4	0.0–7.3	0.7–13.4	0.0–7.3
			Ic/In	51	96^a^	NE	50	NE	20	NE
			CI^I^	29–126	72–137	NC	20–50	NC	NC	NC
	June	38	P	28.9	44.7^b^	5.3	5.3	2.6	2.6	2.6
			CI^P^	16.7–45.3	29.6–60.6	0.9–18.0	0.9–18.0	0.1–14.0	0.1–14.0	0.1–14.0
			Ic/In	38	76^ab^	20	20	20	20	+
			CI^I^	27–56	46–156	NC	NC	NC	NC	NC
	July	39	P	23.1	38.5^b^	2.6	2.6	0.0	0.0	5.1
			CI^P^	12.3–38.4	24.1–55.2	0.1–13.6	0.1–13.6	0.0–8.6	0.0–8.6	0.9–17.5
			Ic/In	722	68^b^	100	140	NE	NE	+
			CI^I^	36–2,890	31–159	NC	NC	NC	NC	+
	August	48	P	31.2	33.3^b^	8.3	0.0	0.0	0.0	4.2
			CI^P^	19.6–45.8	21.1–47.9	2.9–19.6	0.0–7.8	0.0–7.8	0.0–7.8	0.7–14.3
			Ic/In	260	43^b^	20	NE	NE	NE	++
			CI^I^	52–677	25–77	NC	NC	NC	NC	++
	September	57	P	29.8	45.6^b^	8.8	1.8	1.8	0.0	5.3
			CI^P^	19.1–42.9	33.2–58.8	3.5–19.1	0.1–9.3	0.1–9.3	0.0–6.6	1.4–14.6
			Ic/In	72	202^a^	472	40	20	NE	+
			CI^I^	42–121	111–324	128–616	NC	NC	NC	(+)–(++)
	October	54	P	31.5	40.7^b^	5.6	0.0	3.7	0.0	7.4
			CI^P^	20.2–45.3	28.4–54.7	1.5–15.5	0.0–6.9	0.7–12.7	0.0–6.9	2.6–17.4
			Ic/In	648	145^a^	27	NE	50	NE	+++
			CI^I^	74–3,480	76–235	20–33	NC	20–80	NC	(+)–(+++)

1 P – prevalence of infection in %; CI^P^– 95% confidence interval of prevalence (lower and upper limits); Ic/In – mean faecal oocyst/egg output intensity, given as the number of coccidial oocysts/nematodal eggs per g of faeces (OPG/EPG); CI^I^– 95% confidence interval of the population means (lower and upper limits); NC – not calculated (only one infected host, or EPG value constant); NE – not estimated (no infected host in the sample); (+), (++) and (+++) – scale of faecal egg output for *Moniezia benedeni*: (+) <10 eggs, (++) 10–30 eggs, (+++) >30 eggs;

a, b, e, f – within items, different superscript letters between particular infection rates (P or Ic/In) in the same column indicate significant difference at P-value < 0.05 (^a, b^), or P-value < 0.001 (^e, f^)

**Table 2.** Proportion of particular *Eimeria* species (%) infecting organic beef cattle, based on a total of n = 600 oocysts (150 oocysts from each herd) identified after sporulation

Species of *Eimeria*	Herd				Total
	A	B	C	D	
*E. bovis*	21.3	52.0	8.0	67.3	37.1
*E. zuernii*	38.7	16.7	65.3	12.0	33.1
*E. alabamensis*	0.7	ND	ND	3.3	1.0
*E. auburnensis*	6.7	ND	2.7	3.3	3.2
*E. ellipsoidalis*	10.0	16.0	12.0	8.0	11.5
*E. bukidnonensis*	3.3	ND	3.3	ND	1.7
*E. pellita*	2.0	12.0	2.0	0.7	4.2
*E. subspherica*	3.3	ND	2.7	1.3	1.8
*E. cylindrica*	0.7	3.3	ND	2.0	1.5
*E. canadensis*	5.3	ND	ND	1.3	1.7
*E. wyomingensis*	8.0	ND	4.0	ND	3.0
*E. brasiliensis*	ND	ND	ND	0.7	0.2

1 ND – not detected

Nematodes of the Strongylida order infected 46.0 (40.2–51.5)% of the examined beef cattle, and the mean faecal egg count reached 113 (88–147) EPG (Table 1). The prevalence varied slightly between herds, from 44.0 (34.4–54.0) to 51.1 (37.0–65.0)%, while the mean EPG value ranged from 48 (33–91) and 56 (42–76) EPG in herds B and A, respectively, to 162 (106–241) and 192 (125–306) EPG in herds D and C, which was found to be a statistically significant difference (P-value < 0.05) (Table 1). The most infected were young animals aged 0.5–1 year and grazing for the first time (P = 69.2 (41.3– 88.7)% and In = 113 (56–184) EPG), although some calves under 6 months of age were also already heavily infected (P = 33.3 (6.3–72.9)% and In = 320 (40–320) EPG). The highest level of infection was observed in May, when there was a significantly higher (P-value < 0.05) prevalence of infection (P = 70.6 (56.9–81.6)%) than in other months, and then at the end of grazing, in September (P = 45.6 (33.2–58.8)% and In = 202 (111–324) EPG). In the middle of summer, the mean faecal egg count was significantly lower (P-value < 0.05) in July (In = 68 (31–159) EPG) and August (In = 43 (25–77) EPG) than in the spring and autumnal months. Either *Ostertagia* sp. or *Haemonchus placei* was the most dominant in the Strongylida species composition, depending on the herd, and each generally was made a greater proportion of all Strongylida than *Trichostrongylus axei, Oesophagostomum* sp. and *Cooperia* sp. (Table 3). Among the remaining strongyle species differentiated by L_3_ larvae, the lowest share was recorded for *Bunostomum* sp. and *Chabertia ovina*, which occurred only in herd C, and for *Nematodirus fillicolis* which was only detected in herd A.

**Table 3.** Genera and species as proportions of all Strongylida (%) in infected organic beef cattle, based on a total of 600 L_3_ infective larvae (150 L_3_ larvae from each herd) identified by larvoscopic examination

Genus or species of Nematoda	Herd				Total
	A	B	C	D	
*Ostertagia* sp.	10.0	28.0	6.0	50.0	23.5
*Haemonchus placei*	65.3	58.0	68.6	13.3	51.3
*Bunostomum* sp.	ND	ND	4.7	ND	1.2
*Trichostrongylus axei*	2.7	3.3	16.6	24.7	11.8
*Cooperia* sp.	ND	8.7	2.7	4.7	4.0
*Oesophagostomum* sp.	20.7	2.0	0.7	7.3	7.7
*Chabertia ovina*	ND	ND	0.7	ND	0.2
*Nematodirus fillicolis*	1.3	ND	ND	ND	0.3

1 ND – not detected

Eggs of *Nematodirus* sp. were found in all but one (herd B) of the examined herds, and a statistically significantly higher level of infection (P-value < 0.05) was found for herd C (P = 11.6 (5.4–21.6)% and In = 313 (118–498) EPG) than for herds B and D (Table 1). Of the other nematodes, the eggs of *Trichuris* sp. were rarely evident in the studied herds, and those of *Aonchotheca* sp. were found even less often (none in herd A), whereas *Strongyloides* sp. eggs were recorded in herds A and C. Threadworm infection was statistically significantly more frequent (P-value < 0.05) in calves (P = 33.3 (6.3–72.9)%) and yearlings (P = 15.4 (2.8–43.4)%) than in 1–2 year-old heifers (P = 0.0 (0.0–8.4)%) and cows (P = 0.9 (0.2–3.2)%).

*Moniezia benedeni* eggs were also observed in all the herds, with the overall prevalence of infection being 4.2 (2.4–7.3)% (Table 1). Statistically significantly (P-value < 0.05), yearlings turned out to be the most vulnerable to cestode infections (P = 23.1 (6.6–52.0)%).

In herd A, the presence of Paramphistomatidae eggs was confirmed by the sedimentation method throughout the grazing season in each of the prepared pooled faecal samples.

### Morphological examination and molecular analyses of paramphistomes

**Fig. 1.**
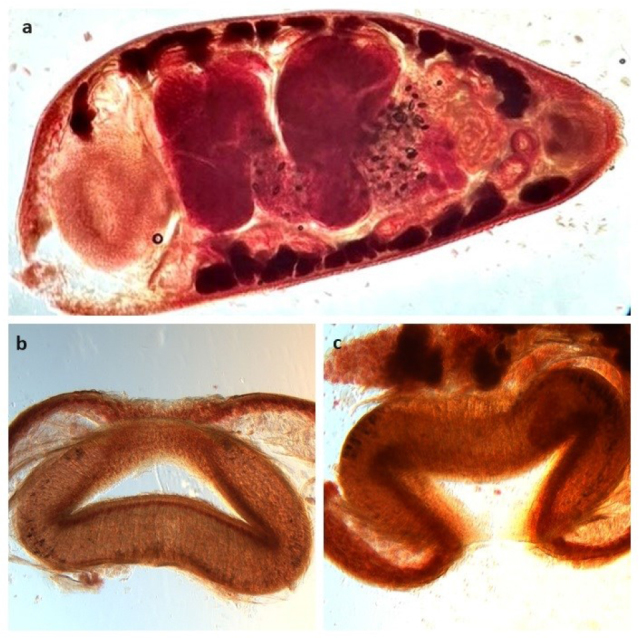
Morphology (sagittal section) of *Calicophoron daubneyi*: a) whole worm; b and c) acetabulum

The examination of the morphological features of adult ruminal flukes collected from a heifer selected in herd A for slaughter already suggested the presence of *Calicophoron daubneyi* (Dinnik, 1962) (Fig. 1). To confirm their species affiliation, selected specimens of these paramphistomes were subjected to molecular analysis. The presence of *C. daubneyi* was confirmed by comparison of the ITS-2 rDNA sequence in the BLAST program, and was the first observation of this trematode species in the country. The sequences were deposited in GenBank under accession Nos PP957466–PP957469, showing 100% similarity to the reference species numbered KP201674.

## Discussion

This is the first finding of *Calicophoron daubneyi* in Poland. Our ITS-2 rDNA sequences were identical to previously published sequences obtained from Irish cattle, indicating the low diversity of this paramphistome in Europe and suggesting a recent invasion from a limited source, with the import of breeding stock. Although this paramphistomid species has occurred in the area of central Europe, having being noted in Bulgaria, Germany, Hungary, Romania and the former Czechoslovakia and Yugoslavia (36), it was not found in Poland when detailed analyses of paramphistomid morphological features were conducted by researchers on Polish samples (13, 45). According to studies carried out in various parts of the country over the last few decades, paramphistomosis has occurred in 2.5–19% of the Polish cattle population, and has been caused by two *Paramphistomum* species: *P. cervi* and *P. ichikawai*, with the first one dominating (13, 28, 30, 32, 39, 45). Of note is that these two paramphistome species were also the only ones found in other domestic (sheep) and wild ruminants (European bison, elk, red deer, roe deer and fallow deer) in Poland (6, 36, 45).

The other digenean trematode, *Fasciola hepatica*, was not observed in the present investigation. Liver fluke infections had been among the most common ones caused by parasites in Poland since the 1960s, but in recent years these infections have been estimated by some authors to be at a prevalence of 6.9 to 8.2% (32, 39), while other studies have not confirmed the occurrence of *F. hepatica* (26, 30, 31). Recent studies have documented that in all of Europe, rumen flukes have been more common than *F. hepatica*, and some authors (3, 30) assumed that this was mainly a consequence of appropriate preventive programmes undertaken against the liver fluke. Current research (15, 22) shows that fasciolosis, which caused quite high economic losses in the past, has apparently been combatted with the extensive use of anthelmintic treatments, whereas *C. daubneyi*, which is resistant to most anthelmintics, has spread throughout Europe. Only lately has it been proved that there is an effective existing flukicide against *C. daubneyi* infections in oxyclozanide (1, 3).

In the geoclimatic conditions of Poland, GIN are the most common parasites of grazing cattle, and the level of infection has remained unchanged over the years. Therefore, the results obtained in our research corresponded with those of previous studies conducted in the country. The research of Pilarczyk *et al*. (31, 32) showed that the prevalence of GIN infection in cows ranged from 12.7 to 67.4%, with a mean faecal egg count from 150 to 455 EPG depending on the management system. Both the prevalence and EPG values, according to Nowosad *et al*. (24, 25), were higher in large herds than in small ones because of higher animal density, and higher in the animals using pastures than in those kept housed. The same authors (25, 26) found the prevalence of GIN infection to be from 38.6–60.3% to even 70–100% in grazing animals, especially in uncontrolled pastured animals (in comparison with those under rotational grazing) and in those using wet pastures. Rotational grazing, in which the quarter is changed every 4–6 days under the environmental conditions for organic farming in this country, is particularly important in preventing animals from becoming infected with the infective stages of nematodes, since the time of 5–8 days is required for the development of nematode larvae to the L_3_ infective stage from the eggs excreted with the host’s faeces to the environment (26). In free grazing conditions, when animals stay in the same area throughout the entire grazing season, they may become infected with a large number of constantly developing infective larvae. In an allied finding, Tomczuk *et al*. (39) noted that with an average Trichostrongylidae prevalence of 35.1%, GIN infection is significantly more common in grazing animals (56.6%) than in those kept exclusively in barns (25.5%), and in beef cattle than in dairy cows, which is likely to be a reflection of the more frequent grazing of beef cattle. However, the level of infection may be high even in housed steers (from 65.8 to 83.7% prevalence and an EPG range of 110–121) if animals managed in a confined system are fed with green forage originating from pastures earlier grazed by cattle (27). In Polish conditions, infective trichostrongylid larvae remain viable on blades of grass in the grazing season for one week, at higher humidity and in less sunlight for up to 3–4 weeks, and in the ground layer for up to 4–5 months. At lower temperatures, they can even overwinter, entering a state of anabiosis and remaining viable for up to 9–12 months (23, 26). Depending on age, GIN infection prevalence ranged from 5.1 to 12% in 5–6-month-old calves, through 21.2% in 6–10-month-old heifers, to 74% in yearlings and 82.7% in 2-year-old animals (24, 31). The faecal egg count in GIN infection cases, which usually was up to 250 EPG in the studies presented, may indicate a constant level of subclinical infection and imply it to have a direct negative impact on productivity, particularly that of the youngest animals. Regardless of the management system, the highest level of infection was observed in herds in the summer months, with a reduction in the autumn and winter months (31). This is not unexpected given the climatic conditions of Poland, where according to the research of Malczewski (19), from September until February over 80% of the nematode population is in the inhibited larval stage, and then rapidly develops to the adult stage, causing the first peak of infections in May. Malczewski (19) also noted the *O. ostertagi* and *H. placei* species presence to be the heaviest.

The annual dynamics of nematode infection in cattle usually exhibits a classic course, having two peaks in EPGs: a higher one in May and a lower one in August (19), although this may vary depending on herd, year or type of pasture (23). In our current investigation, the decrease in the level of infection observed from May to August was sharp, and infection increased only at the end of the grazing season in September and October, despite the lack of deworming. To some extent, this may have been related to the height of grass regrowth, as Wardynski (43) observed that larvae attach to forage at approximately 10 cm above the soil; therefore, grazing above this level may help reduce infection. Polish authors (23) also point out that the differences in the level of infection observed in their studies could be related to the height of the grazed sward, and the lower the height of the grass, the greater the level of infection occurring in the cattle. When optimal conditions are provided, cattle with low EPGs no longer contaminate pastures, and deworming is not required.

In coproscopic and larvoscopic examinations, we found eleven genera of GIN parasites. From the species representing the Trichostrongylidae family, those belonging to the *Ostertagia, Haemonchus, Cooperia* and *Trichostrongylus* genera were most frequently found in grazing animals and were those of major economic importance (28, 32, 39). Although *Oesophagostomum* may also be highly pathogenic (28), Malczewski (19) stated that *O. ostertagi, T. axei* and *H. placei* are of practical significance, as they are dominant in calves. Additionally, a high density of animals, and the attendant significant contamination of barns and paddocks, favours the long-term persistence of pathogenic *Bunostomum* and *Trichuris* infections, which may contribute to long-term diarrhoea in cattle (39). In our investigation, the age of the animals had bearing on the observed level of infection with particular GIN species. Analogously, the coproscopic studies of Chroust (4) showed the presence of *Nematodirus* sp. eggs mostly in animals of up to one year of age, *Strongyloides* spp. to be rare in calves, and *Trichuris* spp. to be rare in older age groups (2 to 3% in prevalence); *Aonchotheca* spp. were even more rarely recorded. Tomczuk *et al*. (39) further remarked that *Strongyloides* may be temporarily highly pathogenic to calves, and that suckler cows are important carriage hosts for these parasites. Obviously, the problem of parasitic infection increases when animals of different ages are grazed together, because immunity to infection is achieved in cattle only after 18–20 months of life; hence, older animals are the source of infection for young, unimmunised ones (25).

Apart from GINs, coccidia of the *Eimeria* genus are very common parasites in Poland’s cattle herds. They affected 93.0% of farms and were found more frequently on larger farms (97.4%) than on smaller ones (89.4%) (17). Calves also became infected with coccidia from older animals that were asymptomatic carriers, including suckler cows, and the main places of infection were moist pastures, poorly maintained barns and paddocks, and natural water reservoirs used for watering (25, 31). This use of natural water sources in pastures could explain the higher coccidia infection in herds A and C (Table 1). The highest level of infection was observed in October, although the intensity of faecal oocyst output reached a maximum value of 722 (36–2,890) OPG in July, which are very similar results to those estimated by Nowosad *et al*. (27). The course of infection was observed to vary greatly between herds (25, 27), with the peak of infection nevertheless being seen trending to late summer and autumn.

When comparing the average status of infection with coccidia, our results are again consistent with those obtained by Nowosad *et al*. (25), whose research showed the prevalence and intensity at the levels of 16.6 to 65.4% and 66 to 377 OPG. They attributed the observed differences to different maintenance conditions in the herds and the examinations of different age groups of cattle. Many authors have described a clear influence of age on the course of coccidia infection, drawing attention to the greater vulnerability of young animals. Coccidiosis most often affects calves aged three weeks to six months, and the level of infection increases with age, reaching a peak at the age of five to seven months. In the research of Pilarczyk *et al*. (29), two-month-old calves were found to be infected with a prevalence of 12.5%, while six-month-old calves’ prevalence rose to 76.5%, that of ten-month-old animals decreased to 49.9%, and in adult cows the infection rate dropped to 27.1%. Other studies by these authors (31) found herd-size-dependent infection rates among adult cows of from 5.5 up to 23.4% (in large herds), with a mean Ic ranging from 100 to 240 OPG, and rates among calves of from 10.0 to 36.8% and Ic of 240 to 540 OPG. In calves with OPG results of above 20,000, Studzińska *et al*. (37) reported the occurrence of clear clinical symptoms, expressed by intense bloody diarrhoea, weakness, dehydration and lack of appetite.

Compared with its course in neighbouring countries, coccidiosis takes a clinical form relatively rarely in Poland, and in most cases it was subclinical in calves (18). Nevertheless, coccidiosis entails economic consequences for beef calf production (29). In Czechia or Germany, the intensity of faecal oocyst output was much higher, reaching 20,000 to 75,000 OPG (4) or even over 100,000 OPG (42), and resulted in clinically observed “summer coccidiosis” in 2–6-month-old calves or 0.5–1-year-old yearlings, clinically manifesting itself as early as 14 days after the start of grazing in first-time grazing animals. Clinical symptoms of coccidiosis result from high environmental contamination with infective stages of highly pathogenic *Eimeria* species, *i.e. E. bovis, E. zuernii* and *E. alabamensis* (38). We observed 12 species of the *Eimeria* genus in our studies, with two of the most pathogenic (*E. bovis* and *E. zuernii*) dominating over the third, *E. alabamensis*, which was seen mostly in one herd. In this herd (farm D), the runs were particularly poorly maintained and were waterlogged.

It is worth mentioning that the species composition of the *Eimeria* genus in domestic cattle is the same as in the free-living European bison populations in Poland. Pilarczyk *et al*. (29) isolated six species of coccidia (*E. bovis, E. auburnesis, E. zuernii, E. ellipsoidalis, E. subspherica* and *E. cylindrica*) from cattle. Demiaszkiewicz *et al*. (6) recognised seven species (*E. bovis, E. pellita, E. zuernii, E. auburnensis, E. cylindrica, E. ellipsoidalis* and *E. brasiliensis*), with *Eimeria bovis* being the most common species in all the bison populations studied. Similarly to the results of our studies, the same species of *Eimeria* infecting the host were revealed by Klockiewicz *et al*. (18) on cattle farms, and by Pyziel *et al*. (33) in free-roaming and captive European bison, *i.e. E. alabamensis, E. auburnensis, E. bovis, E. brasiliensis, E. bukidnonensis, E. canadensis, E. cylindrica, E. ellipsoidalis, E. pellita, E. subspherica* and *E. zuernii*. The most prevalent was *E. bovis* (from 29.7% in bison up to 58.8% in infected calves), while *E. brasiliensis* was the rarest (0.5%).

Coccidia predominate in moist pastures, where infection with helminths, including tapeworms of the genus *Moniezia*, was also observed to be more common by Nowosad *et al*. (25). Our studies revealed the prevalence of *Moniezia* sp. infection to range from 2.1 to 5.8% and be comparable to the prevalence noted by Tomczuk *et al*. (39), while in the research of Nowosad *et al*. (25), it ranged from 1.7 to 22.2% of the animals in the examined herds. It is difficult to compare the results of various authors – not only because of the irregularity in tapeworms’ egg production, but also because of the different flotation methods which they used. Although qualitative methods, *e.g*. modified Wisconsin (16), Willis (39) or Willis–Schlaaf (31, 32) are mainly applied, being more sensitive in detecting *Moniezia* eggs, especially in cases of very low level of infection, quantitative McMaster techniques are also used for this purpose (12, 25).

It would be difficult to limit the level of gastrointestinal parasite infection in the organic herds studied in this research, mainly because the calves and suckler cows are in very frequent contact. Apart from monitoring herds regularly and using appropriate rotational grazing, owners should at least avoid overstocking and overgrazing in their attempts to hold infection in check. With respect to applying proper grazing and pasture management rules, Pilarczyk *et al*. (32) believed that manure, which is the main source of infective parasite forms, should be removed. Of great value might also be avoiding marshy and wet sites, and especially moving cows with their calves as well as heifers from moist parts to higher, drier parts of pastures, and disinfecting barns and paddocks.

## Conclusion

Our study principally reports the finding and presence of *Calicophoron daubneyi* – a highly pathogenic rumen fluke species alien to Poland and a pathogen which deserves special attention not only from the state veterinary service but also from individual farmers and veterinarians in the field. As in other countries in the region (15, 22), its occurrence has been much neglected in recent parasitological research, and it is a lack of awareness rather than the absence of clinical infections which has resulted in failures to report significant problems with helminthiases. With the presence of this species now being known in Poland, it is also important to note that the epizootiology of rumen flukes has been changing (Lymnaeidae besides Planorbidae now being the other intermediate hosts). Also important for risk evaluation is the possibility of parasites being exchanged between related hosts if grazing areas for domestic and wild ruminants overlap. In shared pastures, wild ruminants may locally constitute a permanent reservoir of the infection, as well as even contribute to the spread of flukes to new areas. It should henceforth be regarded as important to regularly monitor grazing cattle herds, because *C. daubneyi* may be transferred from domestic cattle to the natural environment. *Fasciola hepatica*, which shares its intermediate host with *C. daubneyi*, was commonly detected in the forests of north-eastern Poland according to Demiaszkiewicz *et al*. (6), and although no liver flukes were found in European bison in the Bieszczady Mountains, this means that bison may also be threatened. Undoubtedly, further studies should focus on this invasive digenean species which threatens both domestic and wild ruminants.
